# Supplementary material for: Multiple brain activation patterns underlying successful visual short-term memory across the adult lifespan
Source: Imaging Neurosci (Camb). 2026 Jan 8;4:IMAG.a.1074. doi: 10.1162/IMAG.a.1074 (PMC12784220; doi:10.1162/IMAG.a.1074)
Supplement: Supplementary Material [file IMAG.a.1074_supp.pdf]

Supplemental material

Multiple brain activation patterns underlying successful visual short-term memory across the adult lifespan

Michelle G. Jansen[1], Alireza Salami[2, 3, 4, 5], Fernando Ruiz Martínez[1], Daniel J. Mitchell[6], Joukje M. Oosterman[1], Cam-CAN[7], Linda Geerligs[1*]

[1]Donders Institute for Brain, Cognition, and Behaviour, Radboud University, Nijmegen, the Netherlands

[2]Department of Integrative Medical Biology, Umeå University, Umeå, Sweden

[3]Umea Center for Functional Brain Imaging, Umeå University, Umeå, Sweden

[4]Wallenberg Centre for Molecular Medicine, Umeå University, Umeå, Sweden

[5]Aging Research Center, Karolinska Institutet & Stockholm University, Stockholm, Sweden

[6]MRC Cognition and Brain Sciences Unit, University of Cambridge, Cambridge, United Kingdom

[7]Cambridge Centre for Ageing and Neuroscience (Cam-CAN), University of Cambridge and MRC Cognition and Brain Sciences Unit, University of Cambridge, United Kingdom

*Corresponding author: linda.geerligs@donders.ru.nl

Table of content

Supplementary figures......3

Supplementary Figure 1......3

Supplementary Figure 2......4

Supplementary Figure 3......5

Supplementary tables......6

Supplementary Table 1. Model fit indices latent profile analysis......6

Supplementary Table 2. Between-group comparisons of brain module activity......7

Supplementary Table 3. Between-group comparisons of participant characteristics......8

Supplementary Table 4. Within-group associations between task performance and brain module activity......9

Supplementary Table 5. Between-group comparisons of grey matter volumes in each brain module......11

Supplementary Table 6. Between-group comparisons of mean kurtosis in each white matter tract......12

Supplementary figures

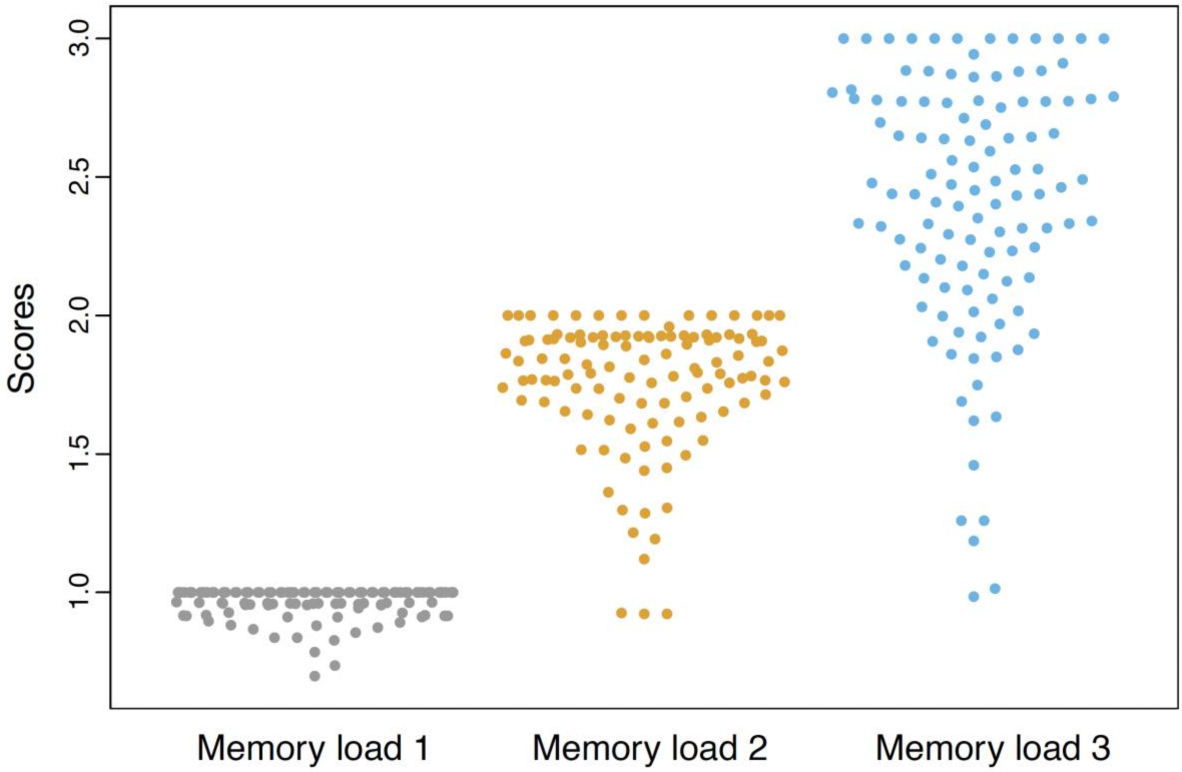

Supplementary Figure 1: The distribution of the number of items in memory at each memory load (load 1-3) as illustrated with beeswarm plots. The number of items in memory for load 1 displayed a ceiling effect, where many participants achieved high performance. This was also present to some extent at load 2. Therefore, the analyses in this manuscript focused on load 3, where the data demonstrated more variation.

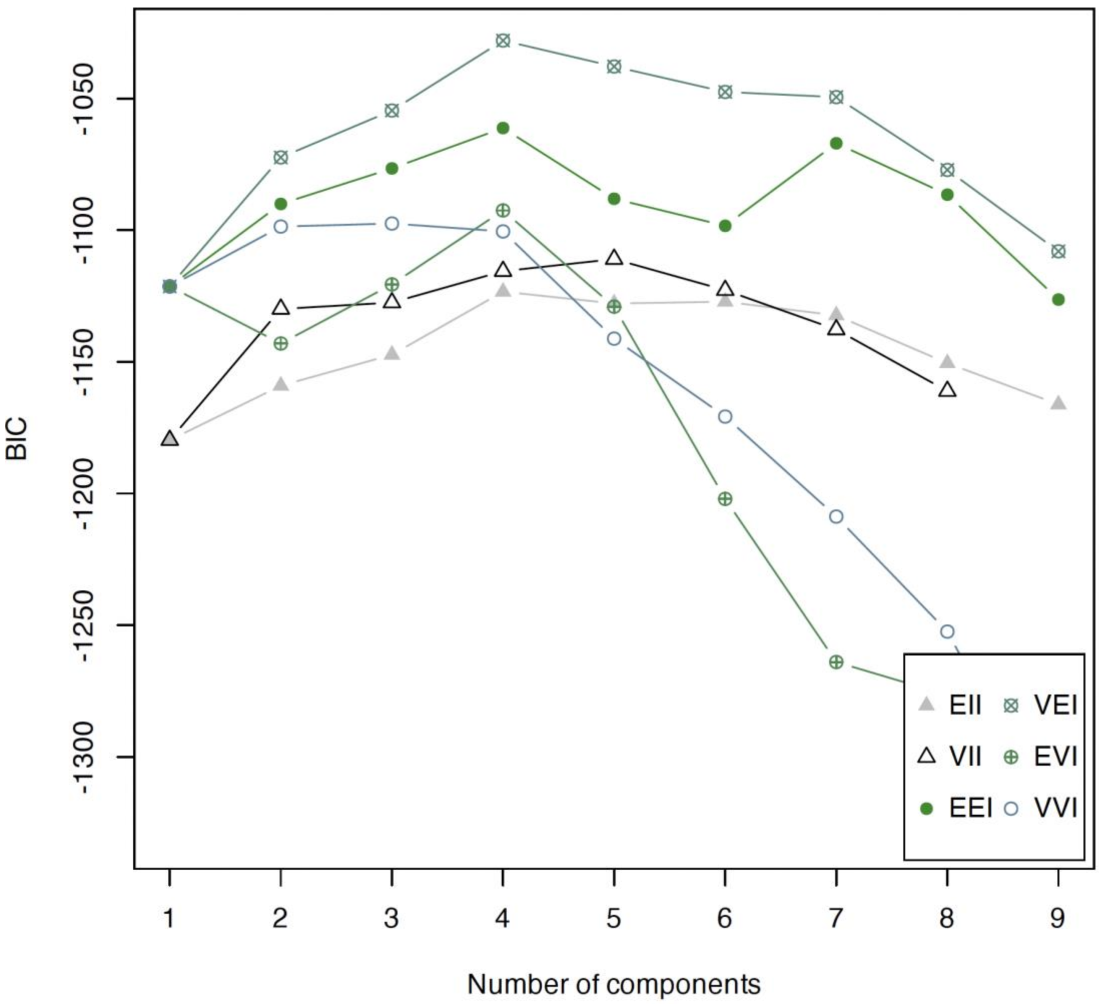

Supplementary Figure 2: Distribution of Bayesian Information Criterion (BIC) values for the different latent-profile models (1-5 clusters) estimated based on the residual activity in each brain network as well as the overall responsivity across all networks. The most optimal BIC value (BIC = -1028) corresponded to a model with 4 latent subgroups of equal shape, variable volume, and a diagonal distribution ("VEI").

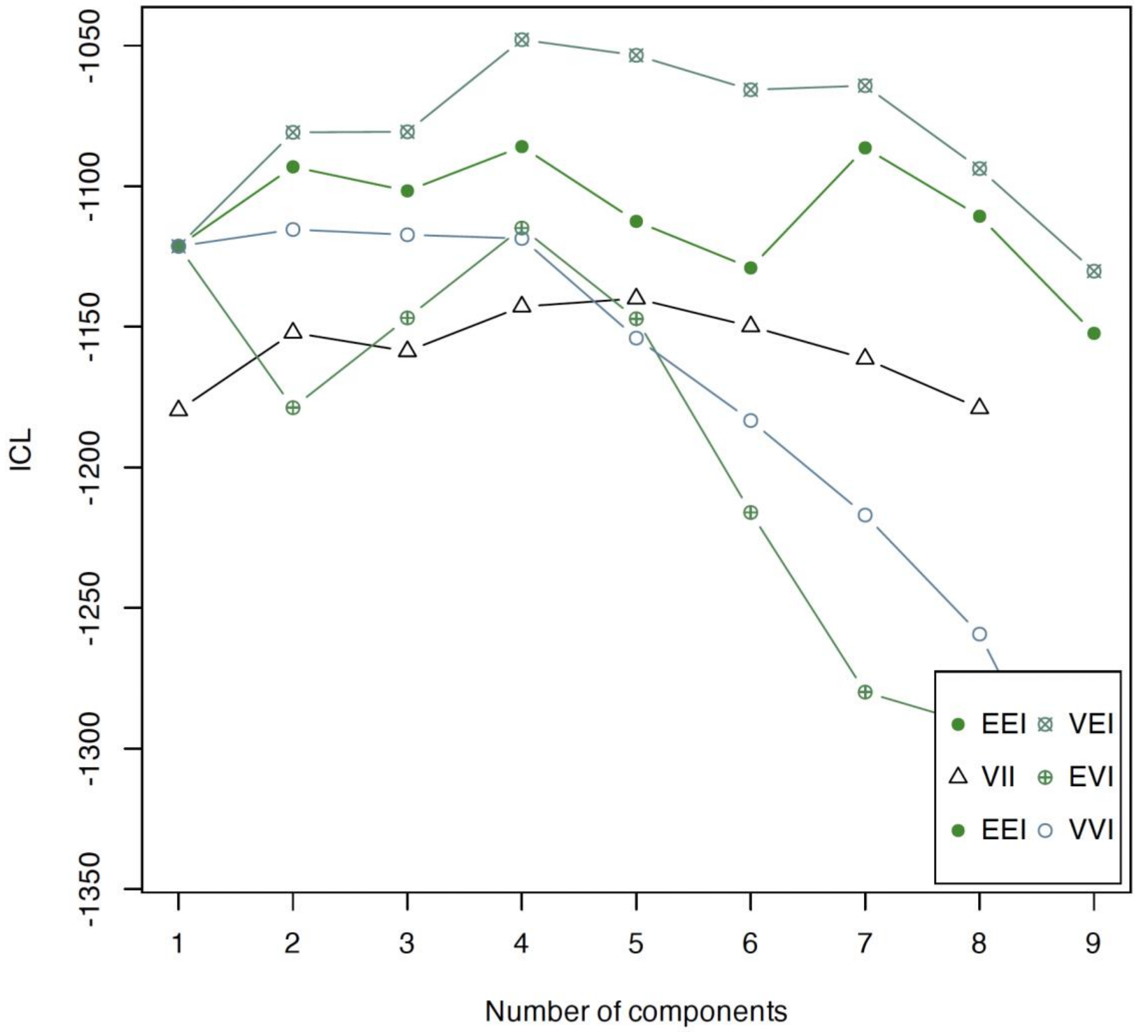

Supplementary Figure 3: Distribution of Integrated Completed Likelihood (ICL) values for the different latent-profile models (1-5 clusters) estimated based on the residual activity in each brain network as well as the overall responsivity across all networks. The most optimal ICL value (ICL = -1048) corresponded to a model with 4 latent subgroups of equal shape, variable volume, and a diagonal distribution ("VEI").

Supplementary tables
Supplementary Table 1. Model fit indices latent profile analysis

Number of classes	df	BIC	ICL	LRT	p-value
1	16	-1121.36	-1121.36		
2	26	-1072.37	-1080.84	96.26	0.001
3	36	-1054.57	-1080.66	65.08	0.001
4	46	**-1028.00**	**-1047.89**	73.85	0.001
5	56	-1037.85	-1054.45	37.42	0.003
6	66	-1047.51	-1065.71	37.61	0.009
7	76	-1049.46	-1064.24	45.33	0.001
8	86	-1077.12	-1093.73	19.61	**0.422**

BIC = Bayesian Information Criterion; ICL = Integrated Completed Likelihood; LRT = Likelihood Ratio Test.

Supplementary Table 2. Between-group comparisons of brain module activity

		No correction applied					Corrected for age				
Brain module	N	F-statistic	p-value	q-value	BF	R^2	F-statistic	p-value	q-value	BF	R^2
**FCM**	**113**	**54.29***	**< 0.001**	**< 0.001**	**3.00e+14**	**0.51†**	**36.92***	**< 0.001**	**< 0.001**	**3.52e+12**	**0.46†**
TPM	113	1.05	0.383	0.44	1.00e-01	0.00	36.79	0.425	0.4859	1.27e-01	0.00
CTM	113	10.92*	< 0.001	< 0.001	1.59e+02	0.15	35.76*	< 0.001	< 0.001	1.78e+02	0.15
**VM**	**113**	**33.93***	**< 0.001**	**< 0.001**	**2.80e+12**	**0.46†**	**37.23***	**< 0.001**	**< 0.001**	**4.26e+11**	**0.44†**
**DAM**	**113**	**20.00***	**< 0.001**	**< 0.001**	**8.16e+05**	**0.28†**	**36.58***	**< 0.001**	**< 0.001**	**6.37e+04**	**0.24**
MM	113	9.45*	< 0.001	< 0.001	2.36e+02	0.15	36.38*	< 0.001	<0.001	1.27e+02	0.14
**DMM**	**113**	**42.56***	**< 0.001**	**< 0.001**	**1.38e+13**	**0.47†**	**37.13***	**< 0.001**	**< 0.001**	**4.72e+11**	**0.44†**
Resp	113	0.77	0.517	0.517	7.00e-02	0.00	38.19	0.512	0.512	6.40e-02	0.00

Results from the Welch's ANOVA and corresponding Bayes Factor (BF) for the alternative hypothesis and Bayesian R^2. Results were considered as significant if both FDR-corrected q-value < 0.05 and BF > 3. Differences were most pronounced for the frontal control module (FCM), visual module (VM), dorsal attention module (DAM), and default mode module (DMM), as reflected by the corresponding values of BF and Bayesian R^2. Results were not affected by age corrections. BF = Bayes Factor; FCM = frontal control module; TPM = temporal parietal module; CTM = cerebellum/thalamus module; VM = visual module; DAM = dorsal attention module; MM = motor module; DMM = default mode module.

*= FDR-corrected q-value < 0.001 & BF > 100.

†$R^2 \geq 0.26$.

Supplementary Table 3. Between-group comparisons of participant characteristics

Continuous variables	N	Df	Df error	F-statistic	p-value	BF	R2
Age	113	3	40.77	2.79	0.05	0.43	0.00
Number of items in memory (load 3)	113	3	44.12	0.45	0.72	0.04	0.00
Memory precision (load 3)	113	3	43.36	1.98	0.13	0.18	0.00
Categorical variables	N	Df	χ^2-statistic	95% CI	p-value	BF	Cramer's V
Sex	113	3	3.43	0.00-1.00	0.32	0.16	0.07
Educational attainment	113	3	4.28	0.00-0.30	0.23	0.26	0.11

Upper part displays the results from the Welch's ANOVA and corresponding Bayes Factor (BF) for the alternative hypothesis and Bayesian R^2. Lower part displays results from the Chi-squared test and corresponding Bayes Factor (BF) for the alternative hypothesis. Results were considered as significant if both FDR-corrected q-value < 0.05 and BF > 3. Notably, educational attainment was statistically compared after dichotomizing the four corresponding categories (university as highest level education vs. the other catgories; A' levels, GCSE grade, none above 16). The between-group comparisons of memory performance were not affected by age corrections.

BF = Bayes Factor.

Supplementary Table 4. Within-group associations between task performance and brain module activity

	Whole sample				Subgroup 1				Subgroup 2				Subgroup 3			
Memory precision (load 3)	*N* = 101				*N* = 35				*N* = 24				*N* = 42			
	R [95% CI]	p-value	q-value	BF	R [95% CI]	p-value	q-value	BF	R [95% CI]	p-value	q-value	BF	r [95% CI]	p-value	q-value	BF
FCM	-0.15 [-0.33; 0.05]	0.136	0.271	0.45	-0.37 [-0.62; -0.04]	**0.030 ***	0.059	2.36	-0.41 [-0.70; 0.01]	**0.045 ***	0.180	1.93	0.12 [-0.19; 0.41]	0.465	0.630	0.30
VM	-0.04 [-0.23; 0.16]	0.718	0.718	0.16	-0.06 [-0.38; 0.28]	0.742	0.742	0.27	0.28 [-0.14; 0.61]	0.191	0.255	0.67	-0.15 [-0.43; 0.16]	0.341	0.630	0.36
DAM	0.17 [-0.03; 0.35]	0.098	0.271	0.58	0.42 [0.10; 0.66]	**0.012 ***	**0.048 ***	4.97	0.30 [-0.12; 0.63]	0.152	0.255	0.79	-0.06 [-0.36; 0.25]	0.696	0.696	0.25
DMM	0.06 [-0.14; 0.25]	0.567	0.718	0.18	0.08 [-0.26; 0.40]	0.637	0.742	0.24	-0.14 [-0.52;0.27]	0.498	0.498	0.38	0.11 [-0.20; 0.40]	0.472	0.620	0.30
Number of items in memory (load 3)																
FCM	0.10 [-0.10; 0.29]	0.316	0.422	0.25	0.24 [-0.10; 0.53]	0.167	0.333	0.63	0.17 [-0.24; 0.54]	0.418	0.679	0.41	-0.10 [-0.39; 0.21	0.531	0.942	0.28
VM	0.03 [-0.17; 0.22]	0.793	0.793	0.16	0.11 [-0.23; 0.42]	0.541	0.541	0.30	-0.09 [-0.32; 0.42]	0.679	0.679	0.33	-0.02 [-0.32; 0.29]	0.917	0.942	0.24
DAM	0.12 [-0.08; 0.31]	0.231	0.422	0.31	0.19 [-0.15; 0.50]	0.264	0.352	0.46	0.18 [-0.24; 0.55]	0.390	0.679	0.43	0.01 [-0.29; 0.31]	0.941	0.942	0.23
DMM	-0.18 [-0.36’ 0.02]	0.073	0.290	0.74	-0.38 [-0.63 – -0.05]	**0.026 ***	0.102	2.67	-0.11 [-0.50; 0.30	0.597	0.679	0.35	0.01 [-0.29; 0.31]	0.942	0.942	0.23

Pearson correlations the associations between visual short-term memory task performance and brain module activity, for each subgroup and corrected for age (i.e., partial correlations). Confidence intervals are standardized. CI = confidence interval; FCM = frontal control module; VM = visual module; DAM = dorsal attention module; DMM = default mode module.

*= FDR-corrected q-value < 0.05 & BF > 3.

Supplementary Table 5. Between-group comparisons of grey matter volumes in each brain module

		Corrected for total intracranial volume					Corrected for total intracranial volume and age				
Grey matter volume	N	F	p	q	BF	R^2	F	p	q	BF	R^2
FCM	113	0.90	0.45	0.45	0.06	0.00	0.15	0.93	0.93	0.03	0.00
TPM	113	5.67**	0.002	0.02	5.10	0.08	2.87*	0.05	0.34	0.72	0.00
CTM	113	3.54*	0.02	0.08	0.89	0.00	2.04	0.12	0.43	0.11	0.00
VM	113	2.97*	0.04	0.08	0.57	0.00	1.04	0.39	0.67	0.07	0.00
DAM	113	2.62	0.06	0.09	0.20	0.00	0.71	0.55	0.77	0.06	0.00
MM	113	3.08	0.04	0.08	0.26	0.00	1.14	0.34	0.67	0.06	0.00
DMM	113	1.78	0.17	0.19	0.13	0.00	0.32	0.81	0.93	0.04	0.00

Results from the Welch's ANOVA and corresponding Bayes Factor (BF) for the alternative hypothesis and Bayesian R^2, corrected for total intracranial volume or additionally for age. To correct for these effects, we regressed the covariate(s) on the variable of interest and used the residuals of that regression. Results were considered as significant if both FDR-corrected q-value < 0.05 and BF > 3. The between-group differences of grey matter volume in the temporal parietal module (TPM) did not remain significant after applying additional age corrections.

BF = Bayes Factor; FCM = frontal control module; TPM = temporal parietal module; CTM = cerebellum/thalamus module; VM = visual module; DAM = dorsal attention module; MM = motor module; DMM = default mode module.

*=uncorrected p-value < 0.05

**=FDR-corrected q-value < 0.05 & BF > 3.

Supplementary Table 6. Between-group comparisons of mean kurtosis in each white matter tract

		No covariates					Corrected for age				
Mean kurtosis	N	F	p-value	q-value	BF	R^2	F	p-value	q-value	BF	R^2
Left anterior thalamic radiation	96	2.95	0.05	0.07	0.88	0.00	1.89	0.15	0.22	0.27	0.00
Right anterior thalamic radiation	96	1.80	0.16	0.21	0.22	0.00	1.28	0.30	0.41	0.10	0.00
Left dorsal cingulate gyrus	96	3.57	0.02	0.06	2.69	0.07	2.69	0.06	0.22	0.92	0.00
Right dorsal cingulate gyrus	95	0.71	0.55	0.58	0.07	0.00	0.49	0.69	0.73	0.05	0.00
Left ventral cingulate gyrus	86	0.12	0.95	0.95	0.04	0.00	0.33	0.81	0.81	0.05	0.00
Right ventral cingulate gyrus	94	1.13	0.35	0.40	0.12	0.00	1.21	0.32	0.41	0.12	0.00
Left cerebrospinal tract	95	5.57 **	0.00	0.02	0.84	0.00	2.06	0.12	0.22	0.15	0.00
Right cerebrospinal tract	94	3.18	0.04	0.07	1.37	0.04	2.81	0.05	0.22	0.56	0.00
Forceps major	96	2.73	0.06	0.09	0.80	0.00	2.13	0.11	0.22	0.32	0.00
Forceps minor	96	3.81	0.02	0.05	1.02	0.01	2.05	0.12	0.22	0.30	0.00
Left inferior frontal-occipital fasciculus	96	4.73 ***	0.01	0.03	5.04	0.09	3.02*	0.04	0.22	1.16	0.02
Right inferior frontal-occipital fasciculus	96	4.08 ***	0.01	0.05	3.49	0.08	2.36	0.09	0.22	0.60	0.00
**Left inferior longitudinal fasciculus**	**96**	**6.71 *****	**0.00**	**0.00***	**28.10**	**0.14**	**5.42 *****	**0.00**	**0.03**	**5.81**	**0.10**
Right inferior longitudinal fasciculus	95	3.40 *	0.03*	0.06	2.18	0.06	2.54	0.07	0.22	0.69	0.00
Left superior longitudinal fasciculus	95	1.91	0.15	0.20	0.23	0.00	1.12	0.36	0.41	0.11	0.00
Right superior longitudinal fasciculus	96	3.18	0.03	0.07	1.00	0.00	1.09	0.37	0.41	0.13	0.00
**Left uncinate fasciculus**	**96**	**6.60 *****	**0.00**	**0.01**	**16.93**	**0.13**	**6.64 *****	**0.00**	**0.02**	**17.32**	**0.13**
Right uncinate fasciculus	96	1.60	0.21	0.25	0.20	0.00	1.89	0.15	0.22	0.28	0.00

Results from the Welch's ANOVA and corresponding Bayes Factor (BF) for the alternative hypothesis and Bayesian R^2, with and without additional corrections for age and average mean kurtosis across all

tracts. To correct for these effects, we regressed the covariate(s) on the variable of interest and used the residuals of that regression. Results were considered as significant if both FDR-corrected q-value < 0.05 and BF > 3. Only the left inferior longitudinal fasciculus and left uncinate fasciculus demonstrated significant differences between-groups after age corrections. BF = Bayes Factor.

*= uncorrected p-value < 0.05; **=FDR-corrected q-value < 0.05

***=FDR-corrected q-value < 0.05 & BF > 3.
